# Management of paroxysmal atrial flutter that occurred in an outpatient prior to dental surgery: a case report

**DOI:** 10.1186/s12903-019-0963-6

**Published:** 2019-12-04

**Authors:** Hajime Shimoda, Tetsu Takahashi

**Affiliations:** Division of Oral and Maxillofacial Surgery, Department of Oral Medicine and Surgery, Tohoku University Graduate School of Dentistry, 4-1 Seiryomachi, Aoba-ku, Sendai, 980-8575 Japan

**Keywords:** Paroxysmal atrial flutter, Heart rate control, Antiarrhythmic agents, Stressful dento-oral surgical ambulatory environment, Emergency care

## Abstract

**Background:**

It is essential to accomplish the appropriate emergency care particularly in patients undergoing stressful dento-oral surgical procedures. Atrial flutter may be induced by sympathetic hypertonia due to excessive mental and physical stress. There is no report regarding dental care in patients with atrial flutter. Herein, we describe a rare case of the antiarrhythmic management in an outpatient who presented with an electrocardiographic finding of paroxysmal atrial flutter before the initiation of the dento-oral surgical procedure.

**Case presentation:**

A 60-year-old male patient was scheduled for a dental extraction. He had a history of angina pectoris, diabetes mellitus, and paroxysmal atrial fibrillation with medication. The preoperative electrocardiogram (ECG) revealed left ventricular hypertrophy and ST-T segment abnormality. Immediately before the dental extraction, II-lead ECG revealed atrial flutter; however, he complained of few subjective symptoms, such as precordial discomfort or palpitation. Observing the vital signs, ECG findings, and the general condition of the patient, low dose diltiazem was immediately administered by continuous infusion in order to control the heart rate and prevent atrial flutter-induced supraventricular tachyarrhythmia. Special attention was paid to prevent any critical cardiovascular condition under a preparation of intravenous disopyramide and verapamil and a defibrillator. The intravenous administration of diltiazem progressively restored the sinus rhythm after converting atrial flutter into atrial fibrillation, resulting in the prevention of tachycardia, and then was found to be appropriate as a prophylactic therapy of tachyarrhythmia.

**Conclusions:**

The present case suggests that it is possible to successfully manage some of such patients using our method during dento-oral surgery which is likely to be associated with mental and physical stress. Therefore, it is essential to accomplish an initial emergency care in parallel to the differential diagnosis of unforeseen serious medical conditions or paroxysmal arrhythmia such as atrial flutter.

## Background

The opportunities for the perioperative systemic management of medically compromised patients with cardiac arrhythmia are steadily increasing in the oral and maxillofacial surgical ambulatory care units. Therefore, it is essential to differentiate possible serious underlying medical diseases and accomplish the appropriate emergency care particularly in patients undergoing stressful dento-oral surgical procedures. Atrial flutter (AFL) may be induced by sympathetic hypertonia due to excessive mental and physical stresses. It is reported that negative emotions such as anxiety or anger increased the likelihood of an atrial fibrillation episode [1, 2], and significantly more arrhythmias occurred before administration of anesthesia than during administration of epinephrine-containing local anesthetics [3]. Before starting the oral surgical procedure for an outpatient, we observed an electrocardiographic finding of paroxysmal AFL. In this report, we discuss the perioperative management of the patient by the administration of antiarrhythmic agents as a prophylactic therapy of tachyarrhythmia.

There is no report regarding the dental care in patients with AFL. The present study patient represents a rare or unusual case of AFL incidence before the dental surgical procedure. Thus, this report contributes to the literature in this area. Further, we think that this paper will be of interest to the readership of the journal because it raises an awareness regarding the importance of perioperative stress-free dental management in patients with arrhythmia.

## Case presentation

A 60-year-old male patient (height: 163 cm, weight: 73 kg) was scheduled for the extraction of the mandibular third molar. He had a history of angina pectoris, diabetes mellitus, and paroxysmal atrial fibrillation with coronary stenting and use of medication, such as diltiazem (100 mg/day), imidapril (5 mg/day), arotinolol (20 mg/day), gliclazide (40 mg/day), pitavastatin (1 mg/day), ticlopidine (200 mg/day), and aspirin (100 mg/day). The preoperative electrocardiogram (ECG) indicated left ventricular hypertrophy and ST-T segment abnormality (leads I, aVL, V5, and V6). The biochemical examination of the blood sample showed hyperglycemia (261 mg/dL) and an increased brain natriuretic peptide level (50 pg/mL).

**Fig. 1:**
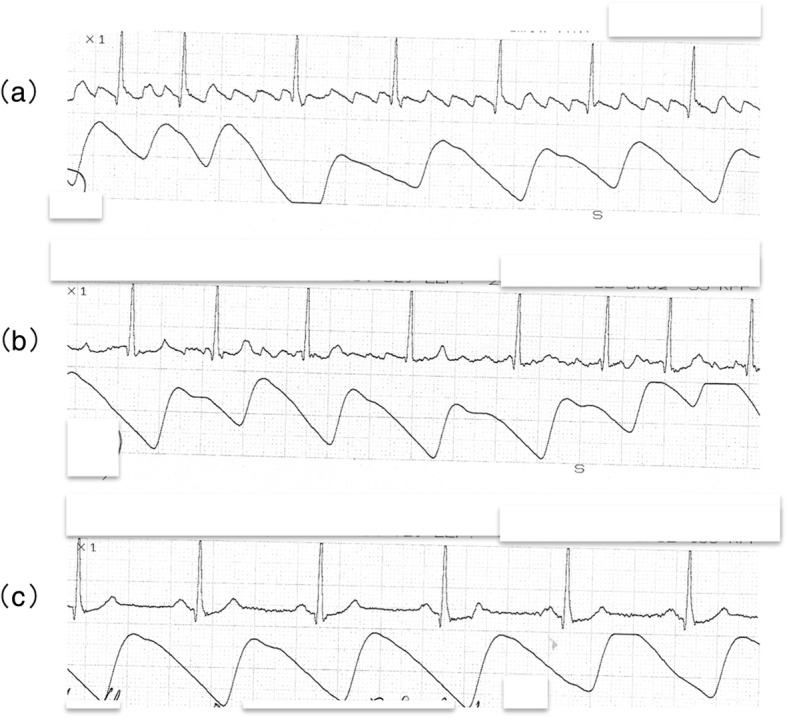
II-lead ECG findings indicating the change from atrial flutter to sinus rhythm (**a**): Atrial flutter, which exhibited the atrioventricular conduction between 2:1 and 4:1, occurred prior to dental surgery. (**b**)/(**c**): Intravenous initial low dose diltiazem (3 μg/kg/min), which was administered by continuous infusion using a syringe pump to prevent cardiovascular disorders such as atrial flutter-induced supraventricular tachyarrhythmia, progressively restored sinus rhythm with success after converting atrial flutter into atrial fibrillation

The analysis of II-lead ECG, noninvasive blood pressure, and oxygen saturation (SpO_2_) before the dental surgery revealed AFL exhibiting the atrioventricular conduction between 2:1 and 4:1 (Fig. 1). At that time, he complained of few subjective symptoms, such as precordial discomfort or palpitation. The cardiorespiratory analysis showed a systolic/diastolic blood pressure of 128/74 mmHg, heart rate of 87 beats per minute (bpm), and SpO_2_ of 98%. With careful monitoring of 12-lead ECG, an intravenous line with saline was rapidly inserted under administration of oxygen (2 L/min) via nasal cannula and low dose diltiazem (3 μg/kg/min), as an antiarrhythmic agent, was immediately administered by continuous infusion using a syringe pump in order to control the heart rate because of the concern about a possible transition into supraventricular tachycardia; this was performed under the auspices of the first author, who is a certified dental anesthesiology specialist. Special attention was paid to prevent critical cardiovascular conditions such as atrial flutter-induced supraventricular tachyarrhythmia under a preparation of intravenous disopyramide and verapamil and a defibrillator for electorical cardioversion. The blood pressure (115–139/68–89 mmHg), heart rate (70–85 bpm), and SpO_2_ (99–100%) were relatively stable with maintenance of consciousness, hemodynamics, and respiratory condition. The intravenous administration of diltiazem progressively restored the sinus rhythm after converting AFL into atrial fibrillation after 30 min and resulted in the prevention of tachycardia. After discontinuing administration of diltiazem, the cardiorespiratory conditions indicated a systolic/diastolic blood pressure of 105/60 mmHg, heart rate of 60 bpm, and SpO_2_ of 99%. No sign of loss of consciousness or cardiorespiratory disturbance such as precordial discomfort, palpitation, or dyspnea was observed. Except sporadic premature supraventricular contractions, the blood pressure (105–133/58–62 mmHg), heart rate (55–65 bpm), and SpO_2_ (96%) were in good control, indicating that the hemodynamic stability was maintained.

We decided to postpone the surgical procedure and consult the cardiovascular internal medicine team because of the concern about a possible recurrence.

## Discussion and conclusions

Patients requiring dental surgery are likely to experience excessive anxiety, fear, and tension even prior to the treatment. This is a widely experienced problem in the dental clinics. Patients with previous aversive painful experiences or unnecessary anxiety or preconception about pain during dental surgery are particularly susceptible to such psychological stresses [2]. In this respect, it is indicated that a frequently postulated mechanism relating the risk of arrhythmia to psychological distress is sympathetic predominance and parasympathetic withdrawal [3, 4]. Furthermore, it is concluded that negative emotions including anger, anxiety, and so forth trigger symptomatic atrial fibrillation [5]. Once the patients consult a dental doctor, they can be exposed to a stressful ambulatory environment of dento-oral surgery in which anxiety would not be sufficiently suppressed [6]. In the light of this viewpoint, it might be also important to recognize the observation that the rare episode in the present patient, although it was preoperative, occurred during the perioporative management closely related to dental care [7]. Therefore, the occurrence of hemodynamic disorders, such as severe hypertension or arrhythmia, due to stress-induced sympathetic hyperactivity or sympathetic/parasympathetic imbalance is of special concern in dental surgery. AFL is often induced by excessive acceleration of the sympathetic nervous activity due to psychosomatic stress; thus, we think that dental surgery patients with episodes of tachyarrhythmia such as the present case with paroxysmal atrial fibrillation should especially be managed by carefully monitoring the circulatory parameters including ECG even during the dento-oral surgical procedures under local anesthesia, because we are concerned about the possible cardiovascular complications involving supraventricular tachycardia or severe unstable hemodynamics.

AFL is characterized by an F wave (sawtooth pattern) that appears as a regular continuous undulation between the QRS complexes, and the negative and positive waves in leads II, III, and aVF are classified as the typical and atypical types, respectively. The atrium during AFL exhibits relatively regular contractions at a rate of 200 to 300 bpm owing to the short circuit in the atrial macroreentrant circuit, which is located (at the cavotricuspid isthmus) along the tricuspid annulus (equivalent to connected portion between the right atrium and ventricle) in the right atrium [8, 9]. As observed in the present study patient, AFL may be a bridge between sinus rhythm and atrial fibrillation, and both arrhythmias can coexist in the same patient [10, 11]. Moreover, the underlying diseases such as ischemic heart disease, diabetes, and hypertension are likely to be latent in AFL [10]. Consequently, We should take into account that the above diseases can be responsible for the risk factors of AFL.

The AFL wave with a dominant positive flutter pattern is considered as an atypical type, which involves a reentrant circuit that rotates around the tricuspid valve annulus in the right atrium in a clockwise direction. The present patient with AFL, which exhibited the atrioventricular conduction between 2:1 and 4:1, may not be aware of palpitation because of a less rapid ventricular rate of 87 bpm at the onset. We guess that if a 2:1 atrioventricular conduction could sustain, the ventricular rate would have exceeded 120 bpm, followed by a complaint of palpitation or chest discomfort. Controlling the heart rate is important to prevent the decrease of cardiac output brought about by the loss of atrial kick owing to tachyarrhythmia. The ventricular rate of the present patient was 87 bpm at the onset, which was not certainly tachycardia. However, we judged that it would be appropriate to prevent cardiovascular disorders such as atrial flutter-induced supraventricular tachyarrhythmia by administration of low dose diltiazem so as not to lead to hypotension and bradycardia, considering a medical history of angina pectoris. We think that the careful administration of low dose diltiazem restored sinus rhythm, leading to minimal reductions in blood pressure and heart rate with satisfaction. Actually, we kept in mind the reference to a cardiologist. On the other hand, because the hemodynamics of the patient was relatively stable, it may be also adequate to refer to a cardiologist firstly, paying great attention to the cardiovascular conditions of the patient.

Regarding the acute medications for AFL, calcium channel blockers and digoxin (as an appropriate agent in patients with cardiac dysfunction) are effective for controlling the ventricular rate or preventing tachyarrhythmia, while class Ia (disopyramide) and class Ic antiarrhythmic agents are beneficial in terminating AFL by overdrive pacing [12]. Primarily, we must consider the risk of 1:1 atrioventricular conduction that may occur due to the prolongation of the AFL cycle by class Ia and Ic antiarrhythmic agents. Therefore, although the patient in the present study had no symptoms related to AFL, we primarily considered controlling the ventricular rate and selectively administered diltiazem, which has less suppressive effect on the cardiac functions. Our aim was to suppress the atrioventricular conduction to prevent supraventricular tachycardia [13, 14]. Verapamil, as an alternative agent, can be advantageous in controlling the ventricular rate; however, it can markedly induce hypotension compared to diltiazem. In the present study, AFL and subsequent atrial fibrillation were successfully reverted to normal sinus rhythm with the administration of diltiazem for a prophylactic therapy of tachyarrhythmia. Accordingly, the initial administration of diltiazem is found to be effective for controlling the ventricular rate and preventing tachyarrhythmia. To avoid any deterioration in hemodynamic stability due to tachyarrhythmia, we think that it is necessary to reduce the atrioventricular conduction preferentially by means of administration of diltiazem as a calcium channel blocker that binds to the L-type calcium channel within the atrioventricular node included in part of the reentrant circuit.

In contrast, it is possible that the anticholinergic action unique to class Ia antiarrhythmic agents may promote the atrioventricular conduction. In this regard, we need to consider restoring the sinus rhythm with Ia antiarrhythmic agents after suppressing the atrioventricular node conductivity and controlling the ventricular rate with calcium channel blockers [11, 15]. Additionally, in the case that medications do not respond to AFL associated with unstable hemodynamics, a defibrillator for electrical cardioversion accompanied by therapeutic anticoagulation should be also prepared with the utmost care and attention [16].

The present case suggests that it is possible to successfully manage some of such patients using our clinical method during the perioperative period in dento-oral surgery which is likely to be psychologically stressful. In addition, as observed from the study findings, sympathetic hyperactivity due to psychological stress in a dento-oral surgical ambulatory clinical environment might increase the intrinsic catecholamines and serve as a causative factor for AFL in patients with ischemic heart diseases, diabetes, and atrial fibrillation. Therefore, it is essential to accomplish an initial emergency care in parallel to the differential diagnosis of unforeseen serious medical conditions or paroxysmal arrhythmia such as AFL.

## Data Availability

Not applicable.

## Abbreviations

AFL: Atrial flutter
ECG: Electrocardiogram
SpO_2_: Oxygen saturation
